# Rv2656c: A Potential Candidate Antigen Associated with Latent Tuberculosis Infection

**DOI:** 10.3390/vaccines14050442

**Published:** 2026-05-15

**Authors:** Yunjie Du, Pu He, Wenrui Dang, Ting Zhou, Yinjuan Song, Xiaoping Li, Yuhao Zhao, Fei Li, Aizhen Guo, Bingdong Zhu

**Affiliations:** 1 State Key Laboratory of Animal Disease Control and Prevention and Lanzhou Center for Tuberculosis Research, Institute of Pathogen Biology, School of Basic Medical Sciences, Lanzhou University, Lanzhou 730000, China; dyj100505@163.com (Y.D.); 18993596041@163.com (P.H.); dangwr2023@lzu.edu.cn (W.D.); zhout011219@163.com (T.Z.); lifei2013666@163.com (F.L.); 2 School of Life Science, Lanzhou University, Lanzhou 730000, China; 3 State Key Laboratory of Animal Disease Control and Prevention, Lanzhou Veterinary Research Institute, Chinese Academy of Agricultural Sciences, Lanzhou 730000, China; syinjuan@126.com (Y.S.); lixiaoping@caas.cn (X.L.); 4 State Key Laboratory of Agricultural Microbiology, College of Veterinary Medicine, Huazhong Agricultural University, Wuhan 430070, China; zhaoyuhao@webmail.hzau.edu.cn (Y.Z.); aizhen@mail.hzau.edu.cn (A.G.); 5 College of Veterinary Medicine, Lanzhou University, Lanzhou Veterinary Research Institute, Chinese Academy of Agricultural Sciences, Lanzhou 730000, China

**Keywords:** *Mycobacterium tuberculosis*, *Mycobacterium bovis*, metabolic enzymes, secreted antigen, latency-associated antigen

## Abstract

**Background/Objectives:** Several subunit vaccines for tuberculosis (TB), such as MVA85A and H4:IC31, have not demonstrated ideal protective efficacy in clinical trials, which may be attributed to their limited antigenic profile and lack of effective latency-associated antigens. In this study, we combined bioinformatics with experimental validation to screen for latency-associated antigens that have immune-protective effects. **Methods:** Highly expressed antigens were identified from models related to latent infections, such as hypoxia and nutritional starvation. Their physicochemical properties and immunogenicity were predicted using online tools such as Expasy-ProParam, IEBD, and VaxiJen. The immunogenicity of these antigens was then evaluated in multiple mycobacterium infection models. Finally, a systematic evaluation of the immune response and protective effects induced by the candidate antigens was performed in a mouse model using intracellular cytokine detection, mycobacterium growth inhibition assays (MGIAs), antibody-dependent cellular phagocytosis (ADCP), and a latent tuberculosis infection (LTBI) mouse model. **Results:** The antigen Rv2656c is highly expressed in the nutritional starvation model and demonstrates strong immunogenicity in both infected humans and cattle. Moreover, Rv2656c exerted a significant inhibitory effect against *Mycobacterium tuberculosis* (*M. tuberculosis*) and *Mycobacterium avium* (*M. avium*) infections in MGIA. The humoral immune response elicited by Rv2656c enhanced the phagocytosis and killing of Mycobacteria by macrophages in vitro. Furthermore, in a mouse model of LTBI established using the attenuated *M. tuberculosis* H37Ra strain, treatment with Rv2656c significantly decreased the bacterial load in the lungs of the mice. **Conclusions:** Latency-associated Rv2656c may serve as an immune-protective antigen, offering potential for the development of novel multi-stage antigen subunit vaccine against TB.

## 1. Introduction

TB is mainly caused by *M. tuberculosis* and *M. bovis*, which belong to the *Mycobacterium tuberculosis* complex (MTBC), and continues to pose a major public health challenge globally. The 2025 Global Tuberculosis Report from the World Health Organization (WHO) indicates that there were around 10.7 million new cases of TB worldwide in 2024 [1]. It is estimated that around a quarter of the world’s population has LTBI. Epidemiological evidence shows that 85% to 90% of new active pulmonary TB cases arise from LTBI [2]. Therefore, effectively preventing and controlling LTBI through vaccination is crucial for reducing the overall burden of TB.

*M. tuberculosis* is an intracellular pathogen that can exist in various forms within macrophages, including both replicative and dormant states. *M. tuberculosis* expresses different antigens at various growth stages. Therefore, selecting the dominant antigens from different growth phases of *M. tuberculosis* as vaccine candidates can broaden the antigen spectrum and induce a comprehensive protective immune response, effectively preventing and controlling TB infection. After vaccination, the antigen-specific T cells induced by the vaccine can recognize the peptide antigens presented by the MHC I and MHC II molecules. Among these, Th1 cells activate macrophages by secreting cytokines such as IFN-γ, promoting the production of antimicrobial substances that further kill the bacteria. Meanwhile, CD8^+^ T cells attack target cells by exerting their cytotoxic effects, further clearing *M. tuberculosis* [3,4]. In addition, the antigen-specific antibodies induced by vaccination may enhance protective immunity against *M. tuberculosis* through mechanisms such as opsonophagocytosis, neutralization, and antibody-dependent cellular cytotoxicity (ADCC) [5].

LTBI occurs when individuals are infected with *M. tuberculosis* but do not exhibit any signs of active TB disease, as confirmed by clinical tests or imaging. During this latent phase, *M. tuberculosis* primarily resides within macrophages [6]. Under certain stress conditions, such as low oxygen levels and nutrient scarcity, these bacteria can enter a dormant state by altering their metabolism and gene expression [7,8]. Researchers have established various models in vitro and in vivo, including the low-oxygen dormancy model [9], the nutrient starvation model [10], and the drug retention model [11], to simulate the stress environments. Through genome-wide analyses of these latent infection models, multiple latency-associated antigens have been identified [12]. Among these, Rv2660c and Rv1813c have been incorporated into the H56:IC31 [13] and ID93/GLA-SE [14,15] subunit vaccines, respectively, demonstrating good immune responses and protective effects in animal models. Additionally, a Sendai virus-based vaccine expressing three latency-associated antigens (Rv2029c, Rv2028c, and Rv3126c) has shown immune protection against both acute infection and LTBI in mouse models [16].

Several TB vaccines have entered clinical trials; only M72/AS01E [17] has shown promising protective effects for various populations, while other vaccines, such as MVA85A [18] and H4:IC31 [19,20], have not achieved ideal outcomes. This may be due to their narrow antigen profiles, which make it challenging to induce comprehensive protection. Although some vaccines have attempted to enhance their protective effects by incorporating latency-associated antigens, clinical trial data have still revealed certain limitations. For instance, the ID93/GLA-SE vaccine was able to increase the number of CD4^+^ T cells in healthy individuals, but only a very small number of Rv1813-specific CD4^+^ T cells were detectable [21]. Similarly, the H56:IC31 vaccine failed to induce a strong Rv2660c-specific CD4^+^ T-cell response in both QuantiFERON-TB (QFT)-negative and QFT-positive individuals [22]. These findings suggest that the main issues currently facing TB vaccines include a limited antigen profile and a lack of effective latency-associated antigens. Therefore, there is still a need to continue exploring new latency-associated antigens that have immune protective effects. 

In this study, latency-associated antigens were systematically screened through a comprehensive literature review, bioinformatics analysis, and experimental validation. Rv2656c was identified as a highly immunogenic, MTBC-specific protective antigen. When administered alongside the Dimethyldioctadecylammonium (DDA) and PolyI: C adjuvant (DP), Rv2656c generated robust immune responses and effectively inhibited Mycobacterial growth both in vitro and in vivo.

## 2. Materials and Methods

### 2.1. Mice and Humans

The mice used in the experiment were 6-to-8-week-old female C57BL/6 mice, purchased from the Animal Center of Lanzhou University (Lanzhou, China). The mice were housed under specific pathogen-free (SPF) conditions. All animal experiments were approved by the Ethics Committee of the Basic Medical Sciences School of Lanzhou University, with the approval number Lzujcyxy20250215.

In a human experiment, a total of 108 individuals from slaughterhouse and farming communities, who displayed no signs of active TB were enrolled, with ages ranging from 18 to 60 years. All participants signed an informed consent form before participation, ensuring they fully understood the study’s purpose, procedures, and potential risks. The study was approved by the Ethics Committee of the Basic Medical Sciences School of Lanzhou University, with the approval number jcyxy20211202. We strictly adhere to data protection regulations to ensure the security of participants’ personal information and data.

### 2.2. Bacterial Strains

The Bacille Calmette-Guérin (BCG, Danish strain) and *M. tuberculosis* H37Ra strains (ATCC 25177) used in this study were generously provided by Fudan University and the Lanzhou Institute of Biological Products, respectively. The *M. avium* strain (ATCC 700898) was purchased from the China Center of Industrial Culture Collection (CICC, Beijing, China). To activate the frozen BCG, H37Ra, and *M. avium* strains, they were first streaked in three zones on 7H10 agar (supplemented with OADC). After 14 days, the cultures were transferred to a 7H9 medium (supplemented with OADC) for further enrichment. Following an additional two weeks of incubation, the cultures were centrifuged at 6000 revolutions per minute (rpm) for 10 min to collect the bacteria. The bacterial pellet was resuspended in PBS, mixed with 50% glycerol, and stored at −80 °C. The frozen bacterial strains were then thawed and used to count the colonies on a 7H10 solid medium.

### 2.3. Screening of M. tuberculosis Latency-Associated Antigens

**Figure 1:**
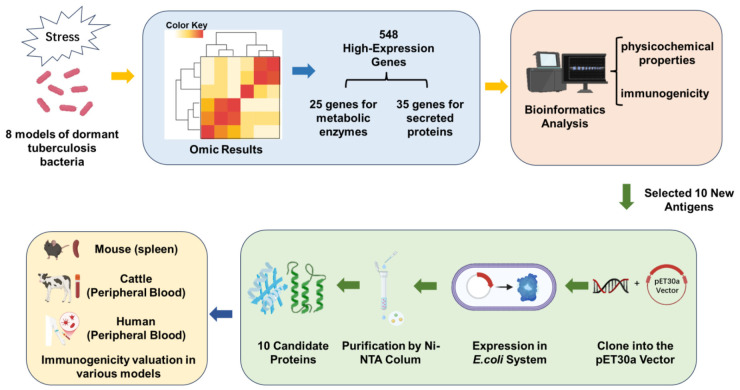
The screening process for latency-associated antigens of *M. tuberculosis.*

By searching the PubMed database using keywords related to tuberculosis latent infection models and tuberculosis dormant bacteria models, models of tuberculosis dormancy induced by a single factor were identified from the search results (conducted from December 2022 to February 2023). As a result, we identified eight models associated with LTBI. These models included the low-oxygen dormancy model [9], the nutrient starvation model [10], the drug retention model [11], the granuloma in vitro model [23], the lysosomal stress model [7], the lipid-rich dormancy model [8], the *M. tuberculosis* proteome in vivo model [24], and the non-replicating persistence (NRP) model [25]. By analyzing their omics results, we further selected antigen genes that encode metabolic enzymes and secretory proteins, which are highly expressed in different models. Then, the antigens’ physicochemical properties and immunogenicity were predicted using various software tools. Finally, the identified antigens were expressed and purified, and their immunogenicity was evaluated in mice, cattle, and humans (Figure 1).

#### 2.3.1. Bioinformatic Prediction

In this study, we obtained the gene sequences and amino acid sequences of the candidate latency-associated antigens from the National Center for Biotechnology Information (NCBI) https://www.ncbi.nlm.nih.gov/; accessed on 13 September 2023). The basic physicochemical parameters, including the isoelectric point and molecular weight of the selected antigens, were analyzed using Mycobrowser (https://mycobrowser.epfl.ch/; accessed on 10 October 2023) and the Expasy-ProParam tool (https://web.expasy.org/protparam/; accessed on 12 October 2023).

To confirm the immunogenicity of the candidate antigens, we systematically predicted the major T-cell and B-cell epitopes of the candidate antigens. The Cytotoxic T Lymphocyte (CTL) epitopes of the antigens were predicted with the IEBD MHC-I prediction tool (https://tools.iedb.org/mhci/; accessed on 5 January 2024). The prediction process utilized the NetMHCpan 4.1 EL algorithm to predict 9-mer peptides targeting Human Leukocyte Antigen (HLA) and mouse H2-b haplotype alleles, with a selection criterion of netmhcpan_el score > 0.85. For predicting the Helper T Lymphocyte (HTL) epitopes of the antigens, the MHC-II epitopes of the antigens were predicted with the IEBD MHC-II prediction tool (https://tools.iedb.org/mhcii/; accessed on 5 January 2024). The prediction process utilized the same NetMHCIIpan 4.1 EL algorithm to analyze 15-mer peptides targeting all HLA and mouse H2-b haplotype alleles, with a selection criterion of netmhcpan_el score > 0.7. The B-cell epitopes of the candidate antigens were predicted with the IEDB (https://tools.iedb.org/bcell/; accessed on 6 January 2024). Additionally, the antigenicity of these candidate antigens was assessed using VaxiJen v2.0 (https://www.ddg-pharmfac.net/vaxijen/VaxiJen/VaxiJen.html; accessed on 6 January 2024), where a score > 0.4 indicates potential good immunogenicity (Table 1).

To analyze the amino acid sequence similarity of these antigens with those from common pathogens, we utilized the Basic Local Alignment Search Tool (BLAST: BLAST+ 2.16.0; https://blast.ncbi.nlm.nih.gov/Blast.cgi; accessed on 22 August 2024) available from NCBI.

#### 2.3.2. Preparation of Single Antigens

The DNA fragments encoding the latency-associated candidate antigens were synthesized by BGI and then cloned into the expression plasmids pET30a and pET32a (only Rv2662). Subsequently, the plasmid was transformed into *Escherichia coli* (*E. coli*) BL21 cells for the expression of the proteins, and the expressed recombinant proteins were purified using a Ni-NTA His column (Cytiva, Washington, DC, USA). Protein expression and purity were evaluated using SDS-PAGE. Before their application in experiments, the protein concentrations were measured with the Pierce BCA Protein Assay Kit (Thermo Fisher Scientific, Waltham, MA, USA).

#### 2.3.3. Assessment of Antigen Immunogenicity in BCG-Immunized Mice

In this study, ten female C57BL/6 mice were randomly assigned to two groups: the BCG group and the PBS group, with five mice in each group. The BCG group received a subcutaneous injection of 5 × 10^6^ CFU of BCG per mouse, while the PBS group received an equivalent volume of 200 µL of PBS administered subcutaneously. Four weeks post-immunization, spleen lymphocytes were isolated from the mice using lymphocyte separation solution and were suspended in an RPMI-1640 medium containing 10% FBS and 100 U/mL penicillin–streptomycin. After counting of the cells, the spleen lymphocytes were seeded at a density of 3 × 10^5^ cells/well into pre-coated ELISPOT plates with anti-IFN-γ (2210007, Dakewe Biotech Co., Ltd., Shenzhen, China) and stimulated with purified candidate antigens (5 µg/mL). The ELISPOT plates were incubated at 37 °C in a 5% CO_2_ environment for 20 h. Subsequent experimental procedures were performed following the instructions outlined in the ELISPOT assay kit manual. Finally, the number of spots formed by the cells (SFCs) was counted using an enzyme-linked spot counter.

#### 2.3.4. Assessment of Antigen Immunogenicity in *M. bovis*-Infected Cattle

The fusion protein Rv3872-CFP-10-ESAT-6 (RCE) is composed of three antigens: Rv3872, CFP-10, and ESAT-6, and was employed to diagnose *M. bovis*-infected cattle [26]. The fusion protein ESAT-6-CFP-10 (EC) contains two differential antigens, ESAT-6 and CFP-10, and was also used for diagnosing *M. bovis* infection in cattle. Peripheral venous blood was collected from the tail veins of cattle that were classified as RCE-IGRA-positive, RCE-IGRA-negative, EC-IGRA-positive, and EC-IGRA-negative. The purified candidate latency-associated antigens (10 µg/mL) were then co-cultured with blood at 37 °C for 20 h. After incubation, centrifugation was performed to collect the supernatant. The level of IFN-γ secretion was measured using an enzyme-linked immunosorbent assay (ELISA) kit (Keqian Biology Co., Ltd., Wuhan, China) to assess the immunogenicity of the antigens.

#### 2.3.5. Assessment of Antigen Immunogenicity in LTBI Individuals

Peripheral blood samples were obtained from these participants for IGRA testing. Based on the results of the IGRA test, the population was divided into IGRA-positive individuals and IGRA-negative individuals. The IGRA-positive group was classified as LTBI, while the IGRA-negative group were considered healthy individuals. The purified candidate antigens (10 µg/mL) were co-cultured with the blood samples at 37 °C. After 20 h, the supernatant was collected by centrifuging the samples. The secretion level of IFN-γ was assessed using an ELISA kit (Thermo Fisher Scientific, Waltham, MA, USA) to evaluate the immunogenicity of the antigens.

### 2.4. Immune Evaluation of the Protein in Mice

#### 2.4.1. Immunization Program

Twenty female C57BL/6 mice were selected and randomly divided into four groups: the PBS group, the DP group, the BCG group, and the Rv2656c/DP group, with five mice in each group. As a positive control, BCG (5 × 10^6^ CFU per mouse) was administered subcutaneously in week 0. For the negative control, PBS and DP adjuvants (200 µL per mouse) were given subcutaneously at 0, 3, and 6 weeks. The DP adjuvant is a combination of DDA and PolyI: C. DDA is a cationic liposome that enhances the delivery and presentation of antigens while promoting Th1-type cellular immune responses. PolyI: C acts as a ligand for the TLR3 receptor, inducing a robust immune response and mitigating the pathological reaction associated with tuberculosis infection [27]. The antigen Rv2656c (5 µg/mouse) was emulsified in the DP adjuvant for immunization and administered subcutaneously at 0, 3, and 6 weeks. (Figure S1). Immune responses from antigen-specific T cells were evaluated at six weeks and twelve weeks post-immunization, respectively.

#### 2.4.2. The Level of IFN-γ and IL-2 Secreted by T Cells in Immunized Mice

At six and twelve weeks following the final immunization, lymphocytes were isolated from the spleens of the mice using lymphocyte separation medium (Dakewe Biotech Company Limited, Shenzhen, China). The isolated lymphocytes were resuspended in RPMI 1640 medium supplemented with 10% FBS and 100 units/mL of penicillin–streptomycin. Subsequently, the lymphocytes were seeded at a density of 5 × 10^6^ cells per well in a 24-well plate. The cells were stimulated with Rv2656c (5 µg/mL) at 37 °C in a 5% CO_2_ atmosphere for 20 h. A protein transport inhibitor (BD, Franklin Lakes, NJ, USA) was introduced midway through the process. After incubation, the cells were collected and surface-stained with anti-CD3-Pacific Blue (BioLegend, San Diego, CA, USA), anti-CD4-FITC (BioLegend, San Diego, CA, USA), and anti-CD8-PerCP-Cy5.5 (BioLegend, San Diego, CA, USA). The cells were subsequently permeabilized using a Cytofix/Cytoperm™ kit (BD, Franklin Lakes, NJ, USA) in accordance with the manufacturer’s instructions and then stained intracellularly with anti-IFN-γ-APC (BioLegend, San Diego, CA, USA) and anti-IL-2-PE (BioLegend, San Diego, CA, USA). The stained lymphocytes were analyzed with a NovoCyte flow cytometer (Agilent, Santa Clara, CA, USA). The gating strategy for flow cytometry is displayed in Figure S2.

### 2.5. In Vitro M. tuberculosis Growth Inhibition Assay

In recent years, the prevalence of nontuberculous Mycobacterial disease has gradually increased globally, with *M. avium* complex (MAC) infections being the most common. Previous studies have reported that the *M. avium* ATCC 700898 strain can persist in the bodies of mice for an extended period [28]. Therefore, we conducted preliminary research using an MGIA experiment to investigate whether the immune response induced by the tuberculosis latent infection candidate antigen Rv2656c could provide broad protective effects, inhibiting the growth of both *M. tuberculosis* and *M. avium*.

The MGIA was employed to assess the capacity of splenocytes from immunized mice to inhibit the growth of *M. tuberculosis* in vitro [29]. The protocol was adapted from previously published methods [30]. In this experiment, 15 female C57BL/6 mice were selected and randomly divided into three groups: the PBS group, the BCG group, and the Rv2656c/DP group, with five mice in each group. As a positive control, BCG (5 × 10^6^ CFU/ mouse) was injected subcutaneously in week 0. For the negative control, PBS (200 µL per mouse) was administered subcutaneously at 0, 3, and 6 weeks. The antigen Rv2656c (5 µg/mouse) was emulsified in the DP adjuvant for immunization and administered subcutaneously at 0, 3, and 6 weeks. At four weeks following the final immunization, splenocytes were prepared at a concentration of 5 × 10^6^ cells in 500 µL of antibiotic-free RPMI 1640 medium, supplemented with 10% FBS, 10 mM HEPES, and 2 mM L-glutamine. *M. tuberculosis* H37Ra and *M. avium* were diluted to 5000 and 50,000 CFU per 500 µL, respectively. In a 24-well plate, 500 µL of the bacterial suspension was mixed with splenocytes to achieve a final volume of 1 mL, then incubated at 37 °C in 5% CO_2_ atmosphere for 4 days. After incubation, cell lysates were serially diluted 10-fold and evenly coated on 7H10 agar enriched with 10% OADC, then incubated at 37 °C to allow for colony growth.

### 2.6. Analysis of Antigen-Specific Antibodies in the Sera of Immunized Mice Using ELISA

Twenty female C57BL/6 mice were selected and randomly divided into four groups: the PBS group, the DP group, the BCG group, and the Rv2656c/DP group, with five mice in each group. Serum was collected via cardiac exsanguination following cervical dislocation at 6 weeks after the final immunization, and antigen-specific IgG, IgG1, and IgG2c levels were measured by ELISA. First, the purified Rv2656c protein (5 µg/mL) was added to a 96-well plate at 100 µL per well and incubated overnight at 4 °C. Next, the liquid in the wells was discarded, and the 96-well plates were washed three times with PBST. Following this, 5% BSA was added, and the plates were incubated at 37 °C for 1 h for blocking. Diluted serum samples (1% BSA) were incubated in the 96-well plates at 37 °C for 2 h. After washing, the plates were incubated with anti-mouse IgG, IgG1, and IgG2c antibodies. Subsequently, 100 µL of 3, 3′,5,5′-Tetramethylbenzidine (TMB) substrate was added to the 96-well plate, and color development was allowed to occur at room temperature for 10 to 15 min. The reaction was stopped by adding 50 µL of 1 M sulfuric acid to each well, and the absorbances were read at 450 nm. The antibody titer was defined as the highest serum dilution with an optical density (OD) value greater than 2.1-fold that of the negative control.

### 2.7. Antibody-Dependent Cellular Phagocytosis of Mycobacteria by Macrophages

The phagocytosis assays were performed as previously described [31]. In this experiment, we selected 16 female C57BL/6 mice and randomly divided them into four groups: the PBS group, the DP group, the BCG group, and the Rv2656c/DP group, with four mice in each group. As a positive control, BCG (5 × 10^6^ CFU/ mouse) was injected subcutaneously in week 0. For the negative control, PBS (200 µL per mouse) was administered subcutaneously at 0, 3, and 6 weeks. The antigen Rv2656c (5 µg/mouse) was emulsified in the DP adjuvant for immunization and administered subcutaneously at 0, 3, and 6 weeks. serum was collected via cardiac exsanguination following cervical dislocation at 6 weeks after the final immunization. FITC dye was solubilized in 0.1 M carbonate/bicarbonate buffer and diluted to 1 mg/mL. The *M. tuberculosis* H37Ra strain was collected and centrifuged at 4500 rpm for 10 min to pellet the bacteria. The bacteria were then mixed with the 1 mg/mL FITC solution and incubated on a shaker at 4 °C for 12 h for staining. After staining, the bacterial pellets were washed with sterile PBS. Next, 1 × 10^5^ *M. tuberculosis* H37Ra cells were co-cultured with 20 µL of serum from immunized mice. This mixture was added to 1 × 10^6^ RAW264.7 macrophages and incubated at 37 °C in a 5% CO_2_ environment for 3 h. Samples were analyzed by flow cytometry. The gating strategy for flow cytometry is shown in Figure S3. Macrophages were collected on day 0 and after a 4-day co-incubation period. The cells were washed three times with sterile PBS and then lysed using sterile water. The resulting lysates were spread onto 7H10 agar plates for colony-forming unit (CFU) counting to assess the bactericidal effect of the macrophages on *M. tuberculosis*.

### 2.8. Mouse Model of LTBI and Immunotherapy Procedure

The therapeutic efficacy of the protein Rv2656c was evaluated in a mouse model infected with the *M. tuberculosis* H37Ra strain [32]. In this experiment, we selected 15 female C57BL/6 mice and randomly divided them into three groups: the PBS group, the DP group, and the Rv2656c/DP group, with five mice in each group. At week 0, mice were intranasally infected with 5 × 10^6^ CFU of the attenuated *M. tuberculosis* H37Ra strain. At weeks 12, 14, and 16, the mice received Rv2656c in an adjuvant of DP, while control mice were administered equivalent volumes of PBS and DP adjuvant on the same schedule. To induce immunosuppression and promote disease reactivation, all mice received intramuscular injections of dexamethasone (0.5 mg/kg) three times once a week, starting at week 20. At week 25, the mice were euthanized, and their lungs were carefully removed to quantitatively evaluate bacterial burden by CFUs.

### 2.9. Data Analysis

The data were analyzed using GraphPad Prism version 8.0 software (GraphPad Software, San Diego, CA, USA). For comparisons between two groups, an unpaired two-tailed Student’s *t*-test was utilized, whereas one-way analysis of variance (ANOVA) was applied for comparisons among multiple groups, followed by Tukey’s post hoc test. A *p*-value of less than 0.05 was considered statistically significant.

## 3. Results

### 3.1. The Screened M. tuberculosis Latency-Associated Antigens

First, we compared the gene expression profiles of latency-associated antigens across eight models of *M. tuberculosis* dormancy. A total of 548 genes were found to be upregulated in these models. Notable examples include well-characterized latency-associated antigens such as Rv1733c, Rv2626c, and Rv2031c.

Previous studies have demonstrated that under conditions characterized by nitrosative stress, oxidative stress, hypoxic conditions, and limited nutrition, *M. tuberculosis* upregulates genes associated with lipid metabolism. These metabolic enzymes may represent promising targets for eliminating dormant *M. tuberculosis*. In this study, we identified 25 genes encoding metabolic enzymes through a literature review, which were among the 548 upregulated genes. Additionally, the secretion of antigens is considered crucial for triggering T-cell-mediated immune responses. Based on the literature, 35 genes encoding secretory antigens were identified in this study [33,34,35]. Ultimately, the immunogenicity and dominant epitopes of the candidate antigens were predicted in silico, resulting in the identification of eleven latency-associated antigens: Rv3131, Rv3290c, Rv0467, Rv1106c, Rv0363c, Rv3546, Rv3568c, Rv2780, Rv2660c, Rv2662, and Rv2656c (Table 2). Among these, Rv2660c is part of the H56:IC31 subunit vaccine.

### 3.2. Rv2656c Exhibited Strong Immunogenicity in Animals Infected with M. bovis

**Figure 2:**
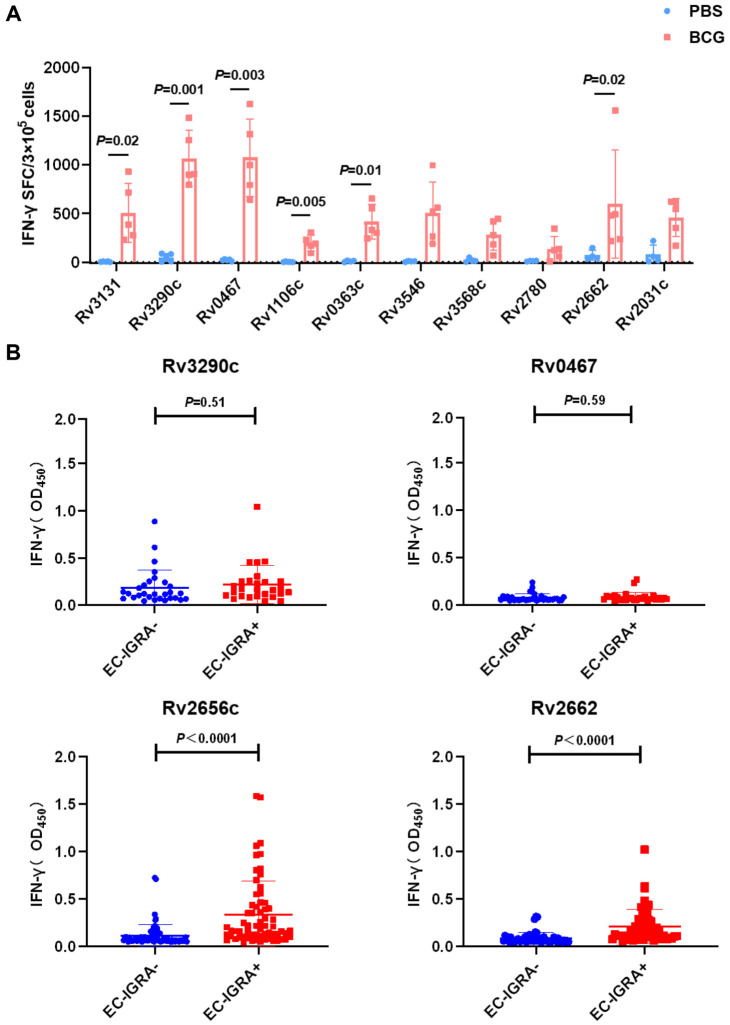
The immunogenicity of latency-associated antigens in animals infected with *M. bovis.* Four weeks after BCG immunization of C57BL/6 mice, splenic lymphocytes were stimulated with purified latency-associated candidate antigens, and the immunogenicity of these antigens was assessed using ELISPOT. (**A**) Statistical analysis of the number of spots corresponding to antigen-specific T cells secreting IFN-γ detected by ELISPOT. Results are expressed as mean ± SD, *n* = 5. (**B**) Statistical analysis of the levels of IFN-γ secretion from peripheral blood from cattle after stimulation with the latency-associated antigens Rv3290c, Rv0467, Rv2656c, and Rv2662. Each dot represents individual cattle, *n* = 138 (64 EC-IGRA-positive cattle and 74 EC-IGRA-negative cattle).

First, we immunized C56BL/6 mice with BCG (5 × 10^6^/mice), and a control group received PBS (200 µL/mice). Four weeks later, splenic lymphocytes were collected and stimulated with purified latency-associated candidate antigens (5 µg /well). The ELISPOT results demonstrated that six candidate antigens—Rv3131, Rv3290c, Rv0467, Rv1106c, Rv0363c, and Rv2662—were effective in stimulating IFN-γ production and showed significant immunogenicity in mice that had been immunized with BCG (Figure 2A and Figure S4). Rv2656c, an antigen located in the RD region of *M. tuberculosis* and absent in BCG, was not assessed for its immunogenicity in this experiment.

Subsequently, we assessed the immunogenicity of the candidate antigens in cattle living in a natural environment. First, we divided the cattle into RCE-IGRA-positive and RCE-IGRA-negative groups using the RCE-IGRA test. After collecting peripheral blood from the cattle via the tail vein, we stimulated the blood samples with the candidate antigens (10 µg/mL). The levels of IFN-γ in the supernatant were measured after 20 h using ELISA. The results indicated that while the antigens Rv3131, Rv0467, Rv1106c, and Rv0363c showed strong immunogenicity in BCG-immunized mice, they did not exhibit good immunogenicity in the wild cattle population (Figure S5). To further assess immunogenicity, we expanded the sample size and evaluated the antigens Rv3290c, Rv0467, Rv2656c, and Rv2662 in cattle. The results revealed that Rv2656c and Rv2662 induced significantly higher IFN-γ secretion in the EC-IGRA-positive cattle compared to EC-negative cattle (Figure 2B).

### 3.3. Rv2656c Induced a High Level of Antigen-Specific IFN-γ Production in LTBI Individuals

**Figure 3:**
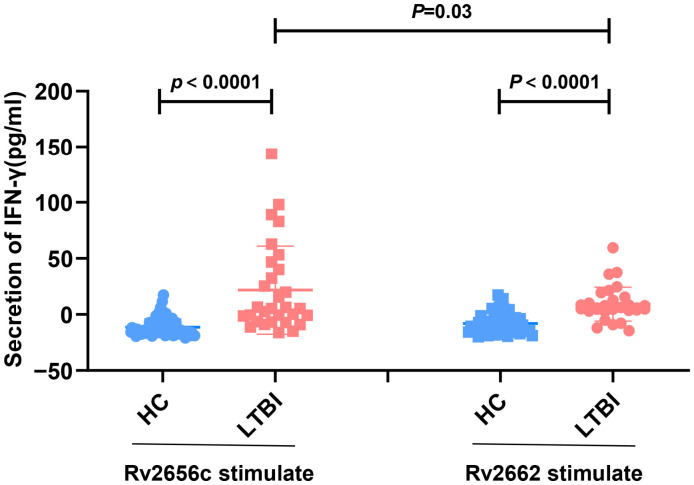
The immunogenicity of latency-associated antigens Rv2656c and Rv2662 in LTBI individuals. The secretion levels of IFN-γ in the culture supernatants were measured using ELISA. Peripheral blood from individuals with LTBI and HC was stimulated with Rv2656c and Rv2662, and the IFN-γ secretion levels were subsequently assessed. Each dot represents a single person. *n* = 108 (30 LTBI individuals and 78 healthy control). Results are presented as means ± SD.

Based on the strong immune response of Rv2656c and Rv2662 in cattle infected with *M. bovis*, we further evaluated their immunogenicity in populations with LTBI. Purified Rv2656c and Rv2662 proteins were used to stimulate peripheral blood samples from these individuals. The results demonstrated that Rv2656c and Rv2662 could significantly induce antigen-specific IFN-γ production in LTBI individuals compared to healthy controls (Figure 3). Among these, the level of IFN-γ secretion induced by Rv2656c is higher than that induced by Rv2662. This indicates that Rv2656c has strong immunogenicity in population with LTBI, warranting further evaluation of its induced immune-protective effects.

It is hypothesized that continuous exposure to common pathogens, such as *E. coli*, may lead to immune tolerance, indicating that antigens with high amino acid sequence homology to these pathogens may not effectively elicit immune memory. From the amino acid homology comparison using NCBI, eight metabolism enzymes (Rv3131, Rv3290c, Rv0467, Rv1106c, Rv0363c, Rv3546, Rv3568c, and Rv2780) showed high homology to common pathogens; however, Rv2656c and Rv2662 showed no homology to these pathogens (Table 3).

### 3.4. Rv2656c in DP Induced Robust Cellular Immune Responses in the Mouse

**Figure 4:**
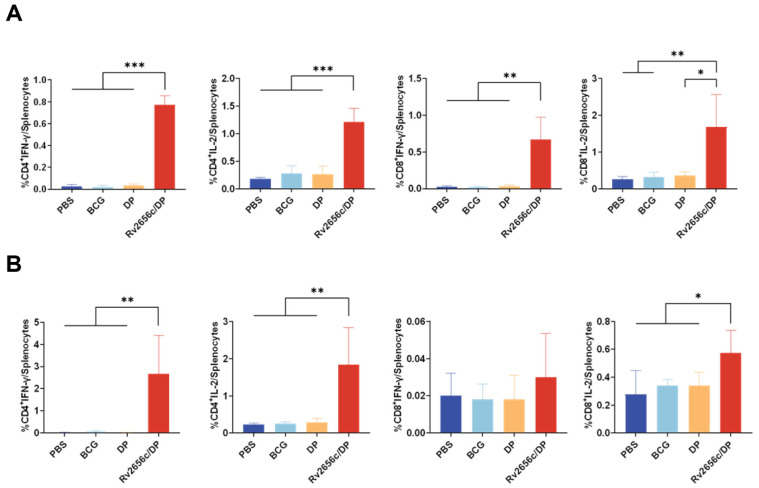
T cells produce IFN-γ and IL-2 in response to antigen-specific stimulation after immunization. At 6 weeks and 12 weeks following the final immunization, splenic lymphocytes were isolated and stimulated in vitro with the Rv2656c antigen for 20 h. The analysis of intracellular cytokine staining was performed using flow cytometry. (**A**) Statistical analysis was performed on the proportion of antigen-specific CD4^+^ IFN-γ^+^,CD4^+^ IL-2^+^, CD8^+^ IFN-γ^+^and CD8^+^ IL-2^+^ Tcells induced by Rv2656c stimulation at 6 weeks after the final immunization. (**B**) Statistical analysis was conducted on the proportion of antigen-specific CD4^+^ IFN-γ^+^, CD4^+^ IL-2^+^, CD8^+^ IFN-γ^+^ and CD8^+^ IL-2^+^ T cells induced by Rv2656c stimulation at 12 weeks following the final immunization. Results are presented as means ± SD, *n* = 5. * *p* < 0.05, ** *p* < 0.01, *** *p* < 0.001.

Six weeks and twelve weeks after the final immunization, we analyzed the immune responses of antigen-specific T cells induced by the Rv2656c/DP using flow cytometry. The results demonstrated that, six weeks after the final immunization, compared to the PBS, BCG, and DP groups, the Rv2656c/DP group elicited a significantly higher presence of IFN-γ^+^ CD4^+^, IL-2^+^ CD4^+^, IFN-γ^+^ CD8^+^, and IL-2^+^ CD8^+^ T cells in the spleen when stimulated with Rv2656c in vitro (Figure 4A and Figure S6). Twelve weeks after the final immunization, the frequencies of antigen-specific CD4^+^ and CD8^+^ T cells producing IFN-γ and IL-2 in mice immunized with Rv2656c/DP were significantly higher than those in the control group. (Figure 4B and Figure S7). This indicates that Rv2656c has strong immunogenicity, and the immune response it induces can last for a relatively long time.

### 3.5. The Immune Responses Induced by Rv2656c Inhibited the Growth of Mycobacteria

**Figure 5:**
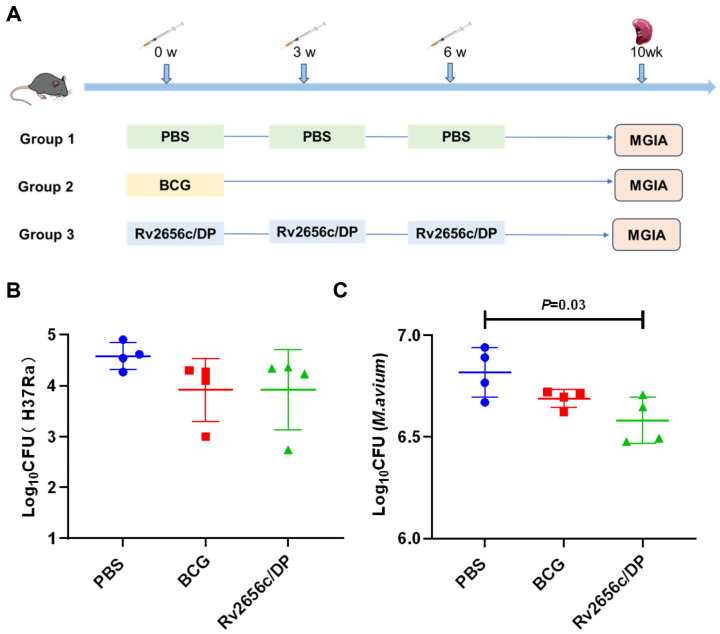
The immune response induced by Rv2656c inhibited the growth of Mycobacteria. The Mycobacteria growth inhibitory effects conferred by splenic lymphocytes from vaccinated and unvaccinated mice were compared using the MGIA method. (**A**) Flowchart of the timeline of the MGIA experiments. (**B**) Results of 5 × 10^6^ splenocytes obtained from mice co-cultured with 5000 CFU H37Ra. (**C**) Results of 5 × 10^6^ splenocytes obtained from mice co-cultured with 50,000 CFU *M. avium*. Results are presented as means ± SD, *n* = 4.

To assess the ability of the immune response induced by Rv2656c/DP to inhibit the growth of Mycobacteria in vitro, the mice in the BCG group received a subcutaneous injection of 5 × 10^6^ CFU of BCG in week 0. Meanwhile, the PBS and Rv2656c/DP groups were immunized subcutaneously at weeks 0, 3, and 6, respectively. Four weeks after the final immunization, spleen cells were isolated and co-cultured with *M. tuberculosis* H37Ra and *M. avium* (Figure 5A). The results showed that the Rv2656c/DP group exhibited significantly greater inhibition of Mycobacterial growth compared to the PBS and BCG control groups. Specifically, the immune response induced by Rv2656c effectively inhibited the growth of both *M. tuberculosis* H37Ra (Figure 5B) and *M. avium* (Figure 5C) in vitro. These findings suggest that Rv2656c has the potential to serve as a protective antigen in subunit vaccines for TB.

### 3.6. Rv2656c Induced Strong Humoral Immune Responses

Six weeks after the final immunization, serum levels of antigen-specific IgG, IgG1, and IgG2c antibodies induced by Rv2656c were measured by ELISA. The results showed that the Rv2656c/DP groups had significantly higher levels of antigen-specific IgG, IgG1, and IgG2c antibodies compared to the PBS, BCG, and DP control groups (Table 4). These findings suggest that Rv2656c effectively induced strong serum antibody responses.

### 3.7. Anti-Rv2656c Antibody Significantly Promoted the Phagocytosis of M. tuberculosis by Macrophages

**Figure 6:**
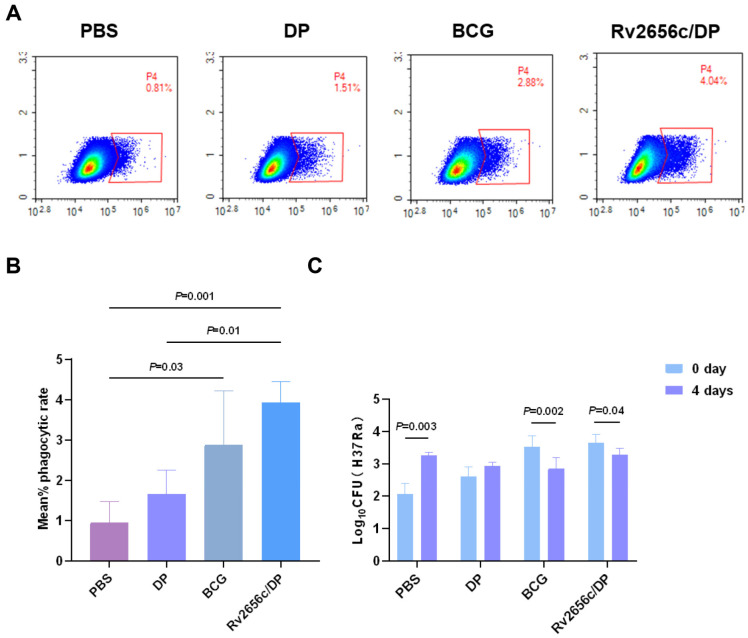
The antibody-dependent phagocytosis and killing of *M. tuberculosis* by macrophages. Determination of the proportion of antibody-dependent macrophage phagocytosis of *M. tuberculosis* using flow cytometry and assessment of the bactericidal efficacy of these macrophages by CFU. (**A**) Flow cytometry analysis of RAW264.7 macrophage phagocytosis of H37Ra in the presence of different antibodies. The above panel is the representative figure. (**B**) Statistical analysis of RAW264.7 macrophage phagocytosis of H37Ra in the presence of different antibodies. (**C**) Quantitative assessment of RAW264.7 macrophage bactericidal activity against *M. tuberculosis* H37Ra, expressed as CFUs. Results are presented as means ± SD, *n* = 4.

To evaluate the contribution of humoral immunity in managing *M. tuberculosis* infection, we evaluated macrophage-mediated antibody-dependent cellular phagocytosis using flow cytometry (Figure 6A). The results indicated that RAW264.7 cells infected with FITC-labeled *M. tuberculosis* H37Ra in the presence of the anti-Rv2656c antibody exhibited significantly higher bacterial uptake compared to those infected in the presence of the PBS and DP control (Figure 6B). Additionally, CFU assays were conducted on *M. tuberculosis* phagocytosed by macrophages at 0 days and 4 days post-infection. The result demonstrated that the anti-Rv2656c antibody not only enhanced the macrophage phagocytosis of *M. tuberculosis* but also significantly improved intracellular bacterial clearance (Figure 6C). This effect was comparable to that observed with the anti-BCG antibody. Collectively, these findings suggest that Rv2656c can induce functional humoral immunity with demonstrable protective capacity against *M. tuberculosis*.

### 3.8. Post-Exposure Administration of Rv2656c Accelerated the Clearance of M. tuberculosis H37Ra

**Figure 7:**
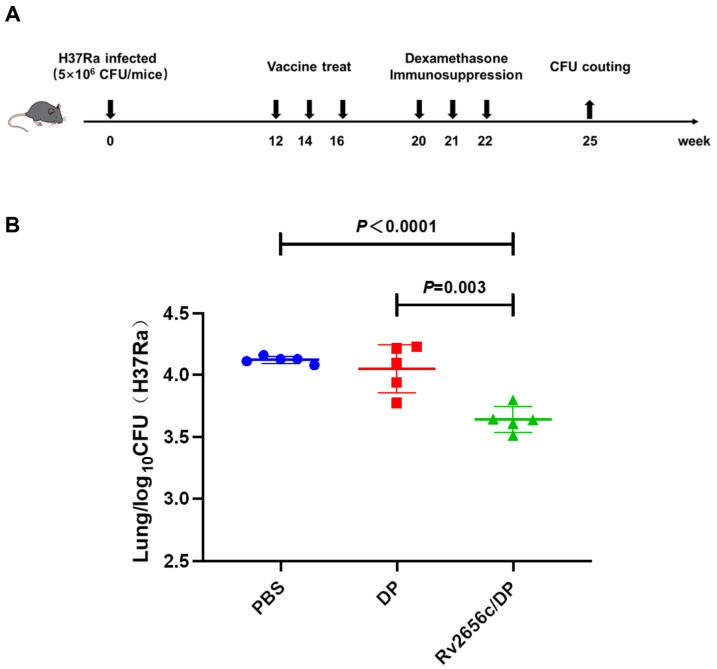
Administration of vaccines enhances *M. tuberculosis* clearance in pre-exposed mice. (**A**) The procedure to establish a H37Ra latent infection model in mice. In week 0, infect the mice with H37Ra. In weeks 12, 14, and 16, subcutaneously immunize the mice with Rv2656c/DP. At the same time, use PBS and DP adjuvants as control groups. In weeks 20, 21, and 22, administer dexamethasone via intramuscular injection to suppress the immune response. In week 25, collect the lungs of the mice for colony counting. (**B**) Statistical analysis of bacterial load in the lungs of mice, presented as Log10. Results are presented as means ± SD. *n* = 5.

To evaluate the immune-protective role of the latency-associated antigen Rv2656c in mice with latent infection, we established a mouse model of latent tuberculosis infection based on a previous report [32]. The mice were treated with Rv2656c/DP to assess their protective effect against *M. tuberculosis*. The CFU count results indicated that compared with the PBS group (4.1 ± 0.03) and DP group (4.05 ± 0.17), the Rv2656c/DP group (3.77 ± 0.2) showed significantly lower CFU counts (Figure 7B). These findings demonstrate that Rv2656c provided significant protection against *M. tuberculosis* H37Ra infection by promoting bacterial clearance and suppressing Mycobacterial replication.

## 4. Discussion

In this study, we identified the latency-associated antigen Rv2656c through in silico screening and experimental validation. Rv2656c demonstrated high immunogenicity in both cattle and humans. Immunization of mice using Rv2656c combined with the DP adjuvant induced strong antigen-specific T-cell and B-cell responses. The specific immune response it elicited effectively suppressed the intracellular replication of Mycobacterium, while the polyclonal antibodies generated enhanced the phagocytosis and killing of the bacteria by macrophages. In addition, in a mouse model of latent infection, immunization with Rv2656c resulted in a significant decrease in the bacterial load in the lungs.

*M. tuberculosis* is an intracellular pathogen that primarily resides and replicates within host macrophages. The host’s defense against intracellular bacterial infections mainly relies on cellular immune responses [36,37,38], especially Th1-type immune responses. Our research indicates that the latency-associated antigen Rv2656c is particularly effective at inducing Th1-type immune responses. Rv2656c stimulates the production of high levels of IFN-γ in the peripheral blood of LTBI individuals and EC-IGRA-positive cattle, demonstrating its strong immunogenicity. In mouse models, immunization with Rv2656c/DP significantly increased the frequencies of antigen-specific CD4^+^ IFN-γ^+^, CD4^+^ IL-2^+^, CD8^+^ IFN-γ^+^, and CD8^+^ IL-2^+^ cells, highlighting their effectiveness in promoting Th1-type immune responses.

It is hypothesized that prolonged exposure to common pathogens may lead to immune tolerance or exhaustion of memory T cells, thereby hindering the establishment of long-term immune protection when highly homologous antigens are introduced. Our analysis, conducted using NCBI to compare candidate antigens related to latent infections with those of common pathogens, revealed that most latency-associated antigens share similar amino acid sequences with these pathogens. Our findings indicate that the latency-associated candidate antigens Rv3131, Rv3290c, Rv0467, Rv1106c, and Rv0363c elicited strong immune responses in BCG-immunized mice; however, they did not effectively induce antigen-specific IFN-γ production in cattle. This discrepancy may be attributed to the fact that the mice were raised in an SPF environment, which is clean and offers limited exposure to common pathogens, thereby enabling them to mount robust immune responses to various antigens. In contrast, cattle that have been exposed to complex environments in the wild for extended periods frequently encounter diverse pathogens, resulting in continuous stimulation from multiple infectious agents. Consequently, when exposed to antigens with amino acid sequences that are highly homologous to those of common pathogens, the cellular immune responses in cattle may be adversely affected, impeding the development of effective immune responses. This implies that using antigens that do not exhibit cross-reactivity with the amino acid sequences of common pathogens may be an effective strategy for the development of subunit vaccines.

In addition to cellular immunity, antibody-mediated immune protection plays a significant role in combating tuberculosis. Previous studies have shown that specific antibodies, such as anti-arabinomannan antibodies induced by BCG vaccination, can enhance ADCP [39]. Our results indicate that Rv2656c not only induces high levels of antigen-specific IgG, IgG1, and IgG2c antibodies in mice but also enhances the phagocytosis and clearance of *M. tuberculosis* by macrophages. Notably, the magnitude of ADCP induced by Rv2656c-specific antibodies is comparable to that elicited by BCG. This suggests that Rv2656c possesses strong immune-protective effects and can be considered a viable candidate antigen for vaccine research and development.

The *M. tuberculosis* attenuated H37Ra strain possesses a highly conserved genome similar to that of the pathogenic strain H37Rv [40,41], which has been used for the preliminary evaluation of TB vaccine efficacy [42,43,44]. Through genetic comparison, we found that the H37Ra strain contains our selected latency-associated candidate antigen Rv2656c. Therefore, in this experiment, we chose to use the attenuated H37Ra strain to evaluate the protective effects of Rv2656c. In subsequent studies, we will further utilize the virulent strain of *M. tuberculosis* to assess the immune-protective effects of Rv2656c.

## 5. Conclusions

In summary, we identified the protective antigen Rv2656c associated with LTBI. This antigen elicited strong, polyfunctional antigen-specific T-cell and B-cell responses in mice. Polyclonal antibodies generated by Rv2656c enhanced macrophage-mediated phagocytosis and intracellular killing of *M. tuberculosis* H37Ra, while Rv2656c-specific immune responses effectively suppressed Mycobacterial replication in vitro. Additionally, Rv2656c provided significant protection by reducing the burden of *M. tuberculosis* H37Ra and suppressing reactivation in mouse models, thereby validating its efficacy as a vaccine antigen. These findings suggest that Rv2656c, as a protective antigen associated with latent infection, holds potential for future TB vaccine development.

## Figures and Tables

**Table 1:** Predictive software and their analytical parameter information.

Predictive Software	Website	Prediction Purpose	Selection Criteria
NCBI	https://www.ncbi.nlm.nih.gov/	Search for antigen genes and protein sequences	/
Mycobrowser	https://mycobrowser.epfl.ch/	Antigenic physicochemical properties	/
Expasy-ProParam tool	https://web.expasy.org/protparam/	Antigenic physicochemical properties	/
MHC-I prediction tool	https://tools.iedb.org/mhci/	CTL dominant epitopes	netmhcpan_el score > 0.85
MHC-II prediction tool	https://tools.iedb.org/mhcii/	HTL dominant epitopes	netmhcpan_el score > 0.7
IEDB	https://tools.iedb.org/main/bcell/	B-cell dominant epitopes	Threshold: 0.50
VaxiJen v2.0	https://www.ddg-pharmfac.net/vaxijen/VaxiJen/VaxiJen.html	Immunogenicity of antigens	Score > 0.4

**Table 2:** Prediction of physicochemical parameters and immunogenicity of latency-associated antigens.

ORF	Gene	Function	Molecular Weight (Da)	Isoelectric Point	CD4 T-Cell Dominant Epitopes (Number)	CD8 T-Cell Dominant Epitopes (Number)	B-Cell Dominant Epitopes (Average Score)	Antigenicity (Score)
Rv3131	Rv3131	NAD(P)H nitroreductase	35,978.4	7.0649	8	8	0.465	0.4185
Rv3290c	lat	L-lysine-epsilon aminotransferase	49,012.1	5.838	12	19	0.441	0.5302
Rv0467	icl1	isocitrate lyase	47,086.6	4.7919	17	25	0.485	0.4787
Rv1106c	Rv1106c	3 beta-hydroxysteroid dehydrogenase/delta 5-->4-isomerase	40,741.6	7.0104	18	20	0.458	0.3639
Rv0363c	fba	fructose-bisphosphate aldolase	36,544.5	5.5853	11	21	0.462	0.5735
Rv3546	fadA5	acetyl-CoA acetyltransferase	41,328.8	5.4226	10	14	0.441	0.4612
Rv3568c	hsaC	extradiol dioxygenase	33,582.3	5.9625	7	20	0.473	0.5344
Rv2780	ald	L-alanine dehydrogenase	38,713.2	6.1772	21	16	0.451	0.5652
Rv2656c	Rv2656c	prophage protein	14,046.7	5.7235	1	8	0.517	0.5182
Rv2660c	Rv2660c	hypothetical protein	7550.32	4.6292	0	3	0.535	0.8648
Rv2662	Rv2662	hypothetical protein	9901.25	8.2212	2	4	0.518	0.3954

**Table 3:** Homology comparison of the amino acid sequence of latency-associated antigens from *M. tuberculosis* with those from common pathogens.

Antigen	*E. coli* (%)	*S. aureus* (%)	*Klebsiella pneumoniae* (%)	*Candida albicans* (%)	*Neisseria* (%)	*Legionella* (%)
Rv3131	99.26%	0	0	0	26.47–32.21	27.81–30.51
Rv3290c	27.75–37.37	21.71–76.56	26.53–37.67	23.24–30.11	23.09–50	22.99–32.23
Rv0467	50.69–91.03	33.33–65.91	45.86–100	35.16	24.76–60.42	24.84–58.12
Rv1106c	25.09–39.44	21.39–56.67	25–65.38	23.41–30.12	21.33–51.72	20.91–34.59
Rv0363c	24.46–78.46	27.83–79.57	25.11–89.74	42.44	24.53–25.9	0
Rv3546	34.13–52.35	26.91–58.9	35.37–44.44	30.75–34.78	35.84–50	32.08–38.24
Rv3568c	38.53–43.36	0	24.27–56.58	24.19	0	0
Rv2780	28.28–66.28	32.61–63.74	29.62–100	0	31.73–52.33	23.46–59.26
Rv2656c	0	0	0	0	0	0
Rv2662	0	0	0	0	0	0

BLAST from the National Center for Biotechnology Information (NCBI) was used to analyze the amino acid sequence similarity to common pathogens.

**Table 4:** The production of antigen-specific IgG, IgG1, and IgG2c antibodies was induced by Rv2656c.

	Groups	Antibody Titers		
		IgG	IgG1	IgG2c
Anti-Rv2656c	PBS	3.05 ± 0.21	1.82 ± 0.15	1.7 ± 0
	BCG	2.96 ± 0.35	1.88 ± 0.24	2.24 ± 0.67
	DP	3.08 ± 0.15	1.88 ± 0.24	2 ± 0.19
	Rv2656c/DP	5.73 ± 0.15 ***	5.73 ± 0.49 ***	5.07 ± 0.29 ***

Six weeks after the final immunization, serum samples were collected from the mice, and the levels of anti-Rv2656c IgG, IgG1, and IgG2c antibodies were evaluated. Results are presented as means ± SD, *n* = 5. *** Rv2656c/DP group vs. PBS, BCG, and DP groups, *p* < 0.001.

## Data Availability

Data are contained within the article and Supplementary Materials.

## Supplementary Materials

The following supporting information can be downloaded at https://www.mdpi.com/article/10.3390/vaccines14050442/s1, Figure S1. vaccine immunization schedule; Figure S2. Flow cytometry gating strategy of the intracellular cytokine staining assay; Figure S3. Flow cytometry gating strategy of phagocytic action assays; Figure S4. Representative ELISPOT images demonstrate the immunogenicity of latency-associated candidate antigens in BCG-vaccinated mice; Figure S5. The level of antigen-specific IFN-γ secretion in the peripheral blood of cattle stimulated by latency-associated candidate antigens; Figure S6. Representative figure of CD4^+^ and CD8^+^ T cells producing IFN-γand IL-2 in response to antigen-specific stimulation 6 weeks after the final immunization; Figure S7. Representative figure of CD4^+^ and CD8^+^ T cells producing IFN-γand IL-2 in response to antigen-specific stimulation 12 weeks after the final immunization.

## Abbreviations

The following abbreviations are used in this manuscript:

ADCC	Antibody-Dependent Cellular Cytotoxicity
ADCP	Antibody-Dependent Cellular Phagocytosis
BCG	Bacillus Calmette–Guérin
BLAST	Basic Local Alignment Search Tool
CFUs	Colony-Forming Units
CICC	China Center of Industrial Culture Collection
CTL	Cytotoxic T Lymphocyte
DDA	Dimethyl Dioctadecyl Ammonium Bromide
DP	DDA and PolyI: C
EdU	5-Ethynyl-2′-Deoxyuridine
ELISA	Enzyme-Linked Immunosorbent Assay
FBS	Fetal Bovine Serum
HLA	Human Leukocyte Antigen
HTL	Helper T Lymphocyte
IEDB	Immune Epitope Database
IGRA	Interferon-γ Release Assay
LTBI	Latent Tuberculosis Infection
MAC	*M. avium* Complex
MGIA	Mycobacterial Growth Inhibition Assay
MHC	Major Histocompatibility Complex
MTBC	*Mycobacterium tuberculosis* Complex
NCBI	National Center for Biotechnology Information
NRP	Non-Replicating Persistence
OD	Optical Density
PBS	Phosphate-Buffered Saline
poly I: C	Polyinosinic-Polycytidylic Acid
QFT	QuantiFERON-TB
rpm	Revolutions Per Minute
SFCs	Spots Formed by the Cells
SPF	Specific Pathogen-Free
TB	Tuberculosis
TMB	3,3′,5,5′-Tetramethylbenzidine
Treg	Regulatory T Cells
