# Supplementary material for: Rv2656c: A Potential Candidate Antigen Associated with Latent Tuberculosis Infection
Source: Vaccines (Basel). 2026 May 15;14(5):442. doi: 10.3390/vaccines14050442 (PMC13211397; doi:10.3390/vaccines14050442)
Supplement: Supplementary file 1 [file vaccines-14-00442-s001.zip › vaccines-4270100-supplementary.pdf]

In Table 2, The MHC alleles included in this study are as follows: HLA-A*01:01, HLA-A*02:01, HLA-A*02:03, HLA-A*02:06, HLA-A*03:01, HLA-A*11:01, HLA-A*23:01, HLA-A*24:02, HLA-A*26:01, HLA-A*30:01, HLA-A*30:02, HLA-A*31:01, HLA-A*32:01, HLA-A*33:01, HLA-A*68:01, HLA-A*68:02, HLA-B*07:02, HLA-B*08:01, HLA-B*15:01, HLA-B*35:01, HLA-B*40:01, HLA-B*44:02, HLA-B*44:03, HLA-B*51:01, HLA-B*53:01, HLA-B*57:01, HLA-B*58:01, H2-Kb, and H2-Db.

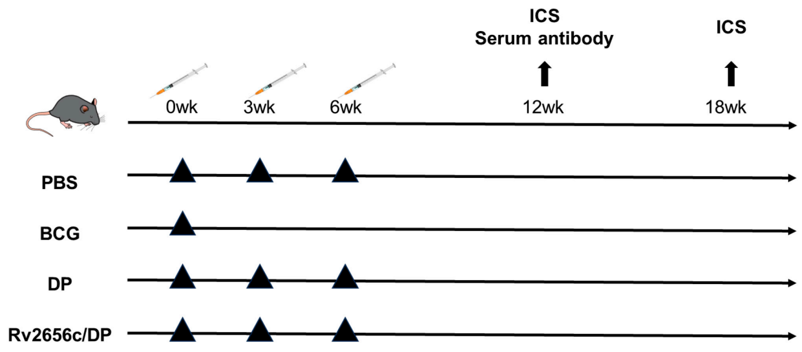

Figure S1. vaccine immunization schedule

The mice were divided into four groups: Rv2656c/DP, DP, BCG, and PBS control. The BCG group received a single injection at week 0, while the other groups were immunized at weeks 0, 3, and 6. The immune responses by antigen-specific memory T cells were assessed at 6 weeks and 12 weeks post-immunization, respectively.

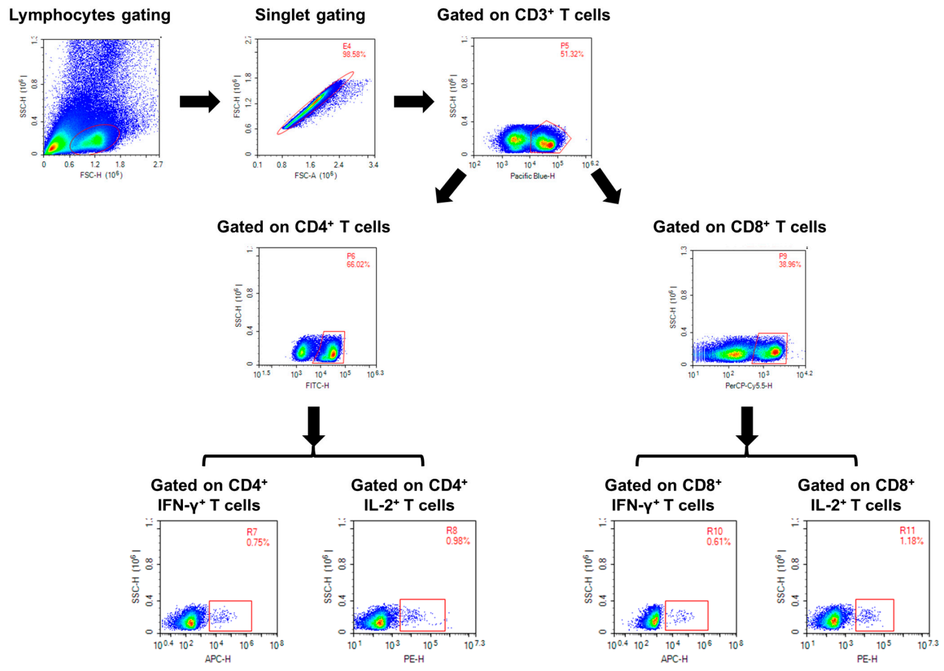

Figure S2. Flow cytometry gating strategy of the intracellular cytokine staining assay

In an intracellular cytokine staining assay, spleen lymphocytes were stained with the anti-CD3, anti-CD4-FITC, anti-IFN-γ-APC, and anti-IL-2-PE. Lymphocytes were first gated by the parameters SSC-H and FSC-H (lymphocytes), and then single cells were gated by the parameters FSC-H and FSC-A (single cells). Finally, $CD4^{+}$ IFN-γ^{+} T cells and $CD4^{+}$ IL-2^{+} T cells were analyzed by flow cytometry.

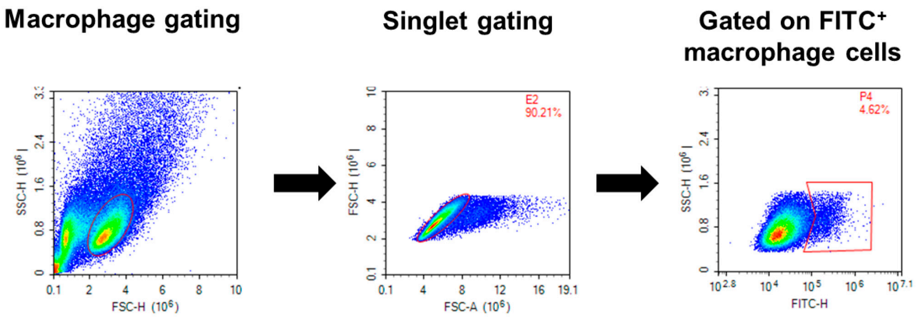

Figure S3. Flow cytometry gating strategy of phagocytic action assays

In phagocytic action assays, macrophages were first gated by the parameters SSC-H and FSC-H (Macrophage), and then single cells were gated by the parameters FSC-H and FSC-A (single cells). Finally, the $FITC^{+}$ T cells were analyzed by flow cytometry.

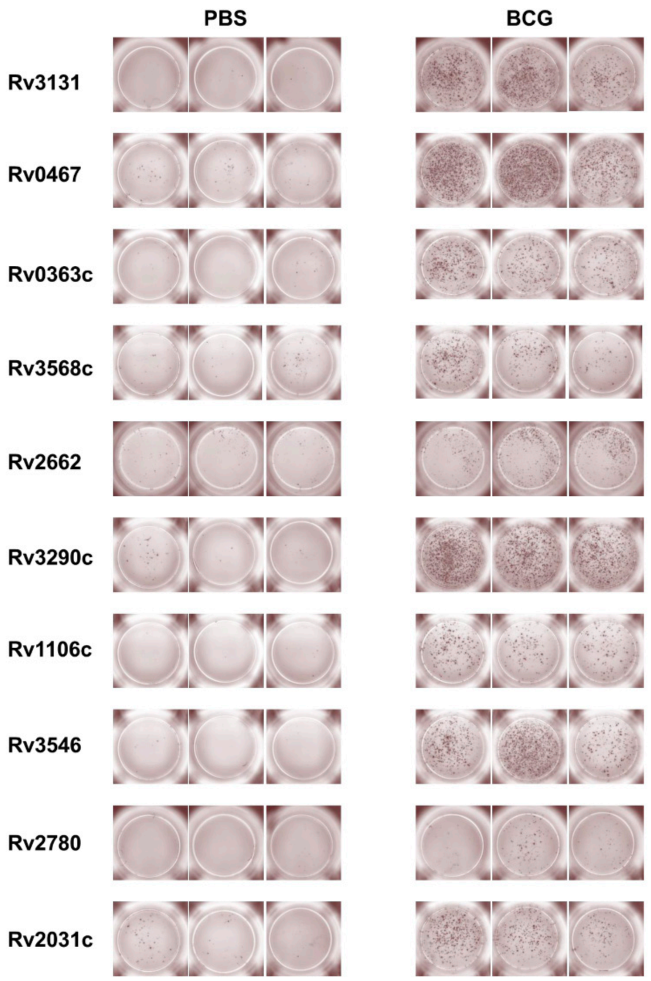

Figure S4. Representative ELISPOT images demonstrate the immunogenicity of latency-associated candidate antigens in BCG-vaccinated mice.

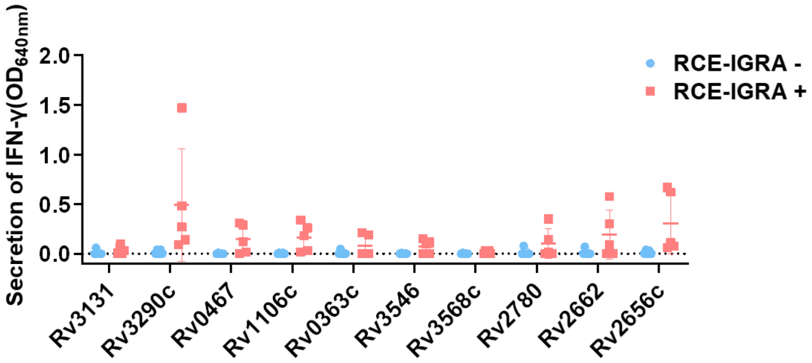

Figure S5. The level of antigen-specific IFN-γ secretion in the peripheral blood of cattle stimulated by latency-associated candidate antigens

Randomly selected cattle herds were divided into RCE-IGRA-positive and RCE-IGRA-negative using RCE-IGRA. Peripheral blood was

collected from the tail vein of the cattle. The peripheral blood of the cattle was stimulated with tuberculosis latency-associated antigen for 20 hours. The supernatant was collected after centrifugation. The IFN-γ secretion level in the supernatant was measured by ELISA. The results were expressed as mean ± SD, *n* = 10 (including 5 RCE-IGRA- cattle and 5 RCE-IGRA+ cattle).

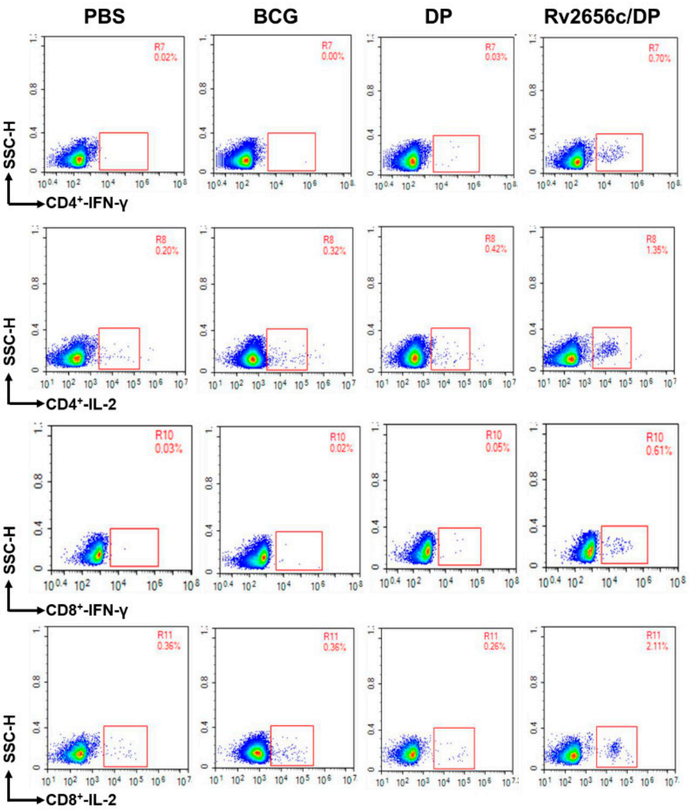

Figure S6. Representative figure of $CD4^+$ and $CD8^+$ T cells producing IFN- γ and IL-2 in response to antigen-specific stimulation 6 weeks after the final immunization.

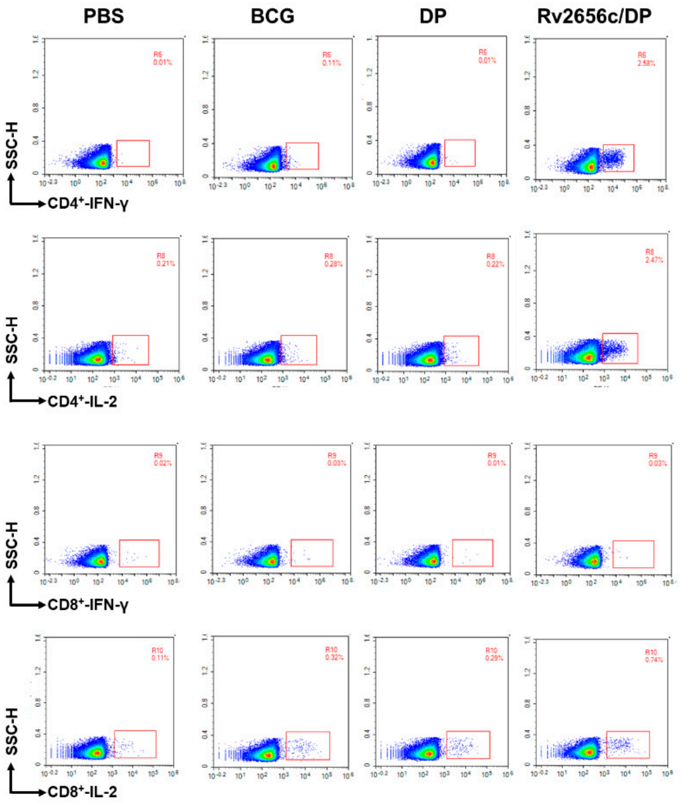

Figure S7. Representative figure of CD4+ and CD8+ T cells producing IFN-γ and IL-2 in response to antigen-specific stimulation 12 weeks after the final immunization.
